# Mental health first aid training for the Chinese community in Melbourne, Australia: effects on knowledge about and attitudes toward people with mental illness

**DOI:** 10.1186/1752-4458-4-18

**Published:** 2010-06-24

**Authors:** Angus YK Lam, Anthony F Jorm, Daniel FK Wong

**Affiliations:** 1 The Centre for Cognitive Behavioural Therapy Education and Training for Chinese People, Department of Social Work and Social Administration, Faculty of Social Science, The University of Hong Kong. Pokfulam, Hong Kong SAR; 2 Orygen Youth Health Research Centre, Centre for Youth Mental Health, University of Melbourne, Parkville, Victoria 3052, Australia; 3 Department of Social Work and Social Administration, Faculty of Social Science, The University of Hong Kong, Pokfulam, Hong Kong SAR

## Abstract

**Background:**

The aim of this study was to investigate in members of the Chinese community in Melbourne the impact of Mental Health First Aid (MHFA) training on knowledge about mental disorders and on attitudes to people with mental illness. The hypotheses were that at the end of the training participants would have increased knowledge of mental disorders and related treatments, and decreased negative attitudes towards people with mental disorders.

**Methods:**

Respondents were 108 participants of three MHFA training workshops for the Chinese community in Melbourne conducted by a qualified MHFA trainer. Participants completed the research questionnaire prior to the commencement of the training (pre-test) and at its completion (post-test). The questionnaires assessed participants' ability to recognize a mental disorder (depression and schizophrenia) described in the vignettes, knowledge about the professional help and treatment, and negative attitudes towards people with mental illness.

**Results:**

Between pre- and post-test there was significant improvement in the recognition of mental disorders, beliefs about treatment became more concordant with health professionals, and negative attitudes reduced.

**Conclusion:**

The MHFA training course for general members of the Chinese community in Melbourne produced significant positive change in the level of mental health literacy and reductions in stigmatizing attitudes. The evidence from this study, together with the accumulated evidence of the benefits of MHFA training in the general Australian community, suggests that this approach should be scaled up to a level where it can have an impact on the whole of the Chinese community in Australia.

## Background

General community members often have some knowledge about handling common physical health problems, whereas knowledge about mental health problems is much less well developed [1]. The prevalence of mental disorders, however, is so high that virtually everyone in the community can be expected to either develop a mental disorder themselves or to have close contact with someone who does [2,3]. Studies on mental health literacy have found that in a number of countries, the general public have poor recognition of mental disorders and beliefs about treatments that often diverge from those of health professionals [4-6]. Finally, there is a widespread stigma on mental disorders which causes an additional burden on sufferers.

### Mental health literacy in Australia

"Mental health literacy" refers to knowledge and beliefs about mental disorders which aid their recognition, management or prevention. It includes the ability to recognize specific disorders; knowing how to seek mental health information; knowledge of risk factors and causes, of self-treatments, and of professional help available; and attitudes that promote recognition and appropriate help-seeking [7]. National surveys were done on Australian population on this area in the years 1995 and 2003. Considerable changes were found in the knowledge and beliefs about mental disorders and their treatment among the Australian public over this 8-year period. These changes involved better recognition of the disorder in a vignette and more positive beliefs about the helpfulness of a range of interventions. In general, these changes involve the public becoming more similar to mental health professionals in their beliefs [8]. Another study, with the South Australia population, examined mental health literacy regarding depression between 1998 and 2004. This research found that fewer respondents used non-specific terms such as psychological problems, nervous breakdown, or work-related problems to describe a person with depression. Instead, there was a greater recognition of depression or depressive symptoms in a vignette as well as in the respondents themselves, with a corresponding increase in treatment seeking and medication use [9].

### Mental health literacy in Chinese community in Australia

Australia is a culturally diverse nation - 43% of Australians were born overseas or have at least one parent who was born overseas [10]. In addition to English, the most commonly spoken languages in the country are Italian, Greek, Cantonese, Arabic, Vietnamese and Mandarin [11]. Research on the mental health literacy of the immigrant population, however, is sparse.

People of Chinese-speaking background make up 3.4% (669,000) of the total Australian population. Overseas and Australian scholars have found that Asians, including Chinese, have a low rate of utilization of mental health services [12,13]. Factors mentioned in the literature as associated with delays in accessing mental health services include knowledge of and beliefs surrounding mental illness and available mental health services [8,14], cultural conceptions of the causes of mental illness [15,16], public and self-stigma [17,18], the tendency to rely on informal networks for support [19], and practical difficulties in accessing services [20].

Only a few studies have explored the mental health literacy of Chinese-speaking people. In studies conducted by Parker and colleagues of Chinese-speaking Australians, the researchers found that (1) Chinese tended to deny depression or express it somatically [21]; (2) Chinese prefer not to view a depressive episode as a disorder or to seek help for a psychological problem [22]; (3) most Chinese confided what they perceived to be private matters only to family members or close friends and (4) many Chinese expected Western medications to provide an instant cure to all kinds of worries, without need for explanation as to how such drugs work [23]. Those results reflect the poor mental health literacy among Chinese-speaking Australians and a study carried out by Wong *et al *[11] supports this claim.

There are also fewer studies exploring the schizophrenia literacy of Chinese-speaking Australian. Klimidis *et al*. [24] found that 47.4% of Chinese-speaking Australians were able to recognize a vignette as depicting schizophrenia when given multiple choice options. 50% of the respondents cited the condition as being related to emotional or mental problems and stress. By contrast, a study by Wong *et al*. [25] found that a much lower percentage of Chinese-speaking Australians (15.5%) was able to identify the vignette as a case of schizophrenia/psychosis when given an open-ended question. This study also found that Chinese-speaking Australians and Japanese were more likely to believe that close family members could be helpful compared to the general Australian population and also expressed more uncertainty about the usefulness or harmfulness of certain medications than the general Australian population.

### Mental Health First Aid

In response to the inadequate mental health literacy in Chinese community in Australia, a suitable training program is required for improving the necessary confidence and skills to provide basic help. Mental Health First Aid (MHFA) training is such a program. It was developed on the basis that people with mental health problems can potentially be assisted by those in their social network [4,26]. The MHFA program, which has now been implemented in many countries, aims to widen the base of people with the knowledge and skills to provide basic assistance to people in the community with mental health problems or in a mental health crisis. The 12-hour training course gives an overview of the major categories of mental health problems, introduces an MHFA Action Plan and applies those actions to problems of depression, anxiety disorders, psychosis and substance use disorders [27]. The course also covers the following mental health crisis situations: how to help a suicidal person, a person having a panic attack, a person who has experienced a traumatic event, a person with psychosis who is perceived to be threatening and a person who has overdosed. In the MHFA training program a structured response, consisting of five actions, is taught. Those actions are:

**1) A**ssess risk of suicide or harm

**2) L**isten non-judgmentally

**3) G**ive reassurance and information

**4) E**ncourage the person to get appropriate professional help

**5) E**ncourage self-help strategies

The initial letters of these actions constitute the mnemonic ALGEE. The MHFA program has more than 750 instructors delivering training across Australia and there are organizations in 14 countries that have adapted the MHFA Australia program for local use. The training course has been evaluated in various settings, with different samples using a range of methods, including randomized controlled trials, uncontrolled trials and qualitative studies. A review of evaluations of MHFA training has highlighted consistent positive benefits in knowledge, behavior, intentions and attitudes of participants [28].

The benefits of MHFA have been demonstrated in several studies with the general Australian community. However, there has been limited evaluation with immigrant communities. The only study to date has been an evaluation with Vietnamese Australians, which showed improvements in knowledge about mental disorders and reductions in stigmatizing attitudes [29]. There has, as yet, been no evaluation of training carried out in a Chinese immigrant community. The aim of this project is to investigate in members of the Chinese community in Melbourne the impact of MHFA training on mental health literacy of the participants' knowledge about appropriate first aid responses and stigmatizing attitudes. Our hypotheses are that at the end of the training participants will have increased knowledge of mental disorders and decreased negative attitudes towards people with mental disorders.

## Methods

### Design

The evaluation involved an uncontrolled pre-test post-test design with three training groups. This study was carried out between November 2008 and January 2009.

### Participants

The participants were recruited through a convenience cluster sampling method in three major areas in metropolitan Melbourne where many Cantonese- and Mandarin-speaking Chinese people live: Box Hill and Doncaster, Monash, and Preston. The recruitment was carried out through social services organizations serving the Chinese population in these three areas. Posters in both traditional and simplified Chinese introducing the research were put up on the notice boards of these organizations. Interested potential participants approached the organizations to register for the program, which was delivered free of charge. Selection criteria included: being (1) aged 18 or above, (2) immigrants of Chinese-speaking background who were living in Melbourne, Australia, and (3) able to read and write basic Chinese.

### Evaluation questionnaire

The participants were asked to complete a questionnaire on their own at the beginning of the program. Oral consent from participants was obtained prior to filling out the questionnaire, after the participants were made aware that participation in the study was voluntary and would not pose any barriers for them to join the program.

This questionnaire covered two categories of information: (1) mental health literacy regarding depression and schizophrenia, and (2) the sociodemographic characteristics of the respondents.

This instrument was adapted from that used by Jorm *et al*., [4] for the study of mental health literacy among Australian samples. This instrument is based around vignettes of a depression (the case of Mary) and one with schizophrenia (the case of John) that are written to satisfy the diagnostic criteria of Diagnostic and Statistical Manual of Mental Disorders, 4th Edition (DSM-IV) and the International Statistical Classification of Diseases and Related Health Problems, 10th Revision (ICD-10).

The participants were asked, "From the information given, what, if anything, is wrong with Mary/John?" (open-ended question) and "Do you think Mary/John needs professional help?" (yes/no). These questions were followed by lists of professionals, treatments and actions that the person in the vignette might use, and participants were asked to rate each of the items as likely to be "Helpful" or "Harmful." "Neither" was also given as a response option.

For depression, there is a professional consensus that GPs, psychiatrists, clinical psychologists, antidepressants, counseling and cognitive behavior therapy are helpful, while for schizophrenia there is a professional consensus that GPs, psychiatrists, clinical psychologists, antipsychotics and admission to a ward are helpful [30]. Thus, for the depression vignette participants received a score from 0 to 6 according to the number of these interventions endorsed as helpful, while for the schizophrenia vignette they scored from 0 to 5. To equalize the range of scores for the two vignettes, they were then converted to percentages.

The questionnaire next assessed stigmatizing attitudes using a social distance scale. Social distance was measured by asking how willing the participant would be to: Move next door to Mary/John; Spend an evening socializing with Mary/John; Make friends with Mary/John; Have Mary/John start working closely with you on a job; Have Mary/John marry into your family. Each question was rated on the following scale: 1. Definitely willing, 2. Probably willing, 3. Probably unwilling, 4. Definitely unwilling. The post-test questionnaire was identical except that the socio-demographic information was excluded. The English version of the scale was translated into Chinese and back translated into English by an experienced professional translator.

### Delivery of the training

The training was delivered by a qualified MHFA instructor, who is a mental health professional (holding both Social Worker and Occupational Therapist qualifications). The instructor was from Hong Kong and had 4 years experience in teaching MHFA there. The adapted MHFA manual was translated into Chinese by a Chinese psychologist. The training program was conducted in Cantonese and was instantaneously translated into Mandarin.

### Statistical analysis

The McNemar test was used for analyzing change in the ability to recognize the disorder and in agreement that the person in each of the vignettes needs professional help. Answers to questions about the helpfulness of various types of intervention and about social distance were scored as scales and changes were evaluated by paired sample t-tests. Statistical data management and analyses of the data were carried out using Statistical Package for Social Sciences (SPSS 18.0 for Windows)

## Results

Table 1 shows the sociodemographic characteristics of the participants. Among 108 participants, 79.7% were women. The age range was from 15 to 73 years with the majority aged from 25 to 54 (55.6%) and a significant proportion (30.6%) of older people. Nearly 50% of the participants had lived in Australia for more than 10 years. Most of them had migrated from Hong Kong and mainland China. Twenty two percent of participants reported themselves to be mental health services users, whereas close to 35% were social services providers. As migrants living in an English speaking country, only 6.48% of the participants rated themselves as having very good English capacity, while over half of them rated themselves as having poor to fair capacity.

**Table 1:** Demographic N = 108

Sex	%
Men	20.4
Women	79.7
	
Age	

15-24	2.8
25-54	55.6
55 or above	30.6
Unknown	11.1
	
Years in Australia	

Less than 5 years	13.9
5-10 years	13.9
11-20 years	32.4
21 or more	17.6
Unknown	22.2
	
Host country	

Mainland China	29.6
Hong Kong	44.4
Vietnam	9.3
Singapore	4.6
Taiwan	2.8
Other	7.4
Unknown	1.9
	
Profile relating to the Use of mental Health services	

Mental Health Services Users	22.2
Family of Mental Health Services Users	12.1
Social Services providers	34.3
Others	24.1
Unknown	7.4
	
Family Income (AUD$)	

0-20000	29.6
20001-40000	19.4
40001-60000	10.2
60001-80000	8.3
80001 or above	12.1
Unknown	20.4
	
Employment Status	

Full Time	19.44
Part Time	26.9
Unemployed	26.9
Retired	20.4
Unknown	6.5
	
Self reported English Capacity	

Very poor	5.6
Poor	13.9
Fair	42.6
Good	30.6
Very Good	6.5
Unknown	0.9

Some of the respondents did not return the post-course questionnaire and thus there were only 84 responses available for pre-post analysis. The percentage correctly recognizing the disorders in the vignettes are shown in Table 2. Only "depression", "schizophrenia" or "psychosis" were considered correct. People were found to have improved in recognizing the disorder from pre-test to post-test (*p *< .001 for depression and *p *= .029 for schizophrenia). Nevertheless, the percentages correctly recognizing the vignettes were far below the results for the Australian population [26].

**Table 2:** Change in the recognition of depression and schizophrenia

	Pre-course%	Post-course%	p*
Depression	19.0	63.1	.000
Schizophrenia	9.5	21.4	.021

*McNemar Test

Table 3 shows the change in percentage of participants believing that professional help would be helpful to the person in vignettes. Even at the pre-test, the percentage was over 90% for both vignettes and there was no significant change at the post-test.

**Table 3:** Change in the agreement of use of professional help of depression and schizophrenia

	Pre-course%	Post-course%	p*
Depression	90.5	96.4	.227
Schizophrenia	94.0	94.0	1.000

*McNemar Test

Table 4 shows the mean scores on scales measuring beliefs about treatment that are concordant with health professionals. A higher score indicates greater agreement with professional beliefs about treatment. There was a statistically significant change from pre-test to post-test for both vignettes (p < .001).

**Table 4:** Change in belief on about treatment.

	Pre	Post	P*
Depression	73.8(24.7)	92.5(15.4)	.000
Schizophrenia	61.4(26.8)	82.9(23.8)	.000

Mean scores (SDs) on scales measuring belief about treatment that are concordant with health professionals

*Paired sample t-test (2-tailed)

Table 5 shows the mean scores on the social distance scale. The score for the schizophrenia vignette, as would be expected, indicated greater social distance than for the depression vignette. After attending the MHFA program, social distance decreased significantly for both groups.

**Table 5:** Social distance on both vignettes

	Pre (SD)	Post (SD)	P*
Depression	10.12 (2.9)	8.8 (2.6)	.003
Schizophrenia	11.7 (3.26)	10.5 (3.3)	.005

*Paired sample t-test (2-tailed)

## Discussion

This is the first evaluation of MHFA training with an immigrant group in a Chinese language. Although the results should be regarded as preliminary, they are encouraging. The study found a number of benefits from MHFA training. The training increased participants' ability to recognize depression and schizophrenia in a vignette, altered beliefs about the helpfulness of treatments for mental health problems and decreased stigmatizing attitudes as measured by social distance.

At pre-test, it appeared that mental health literacy among the Chinese was low, consistent with the findings of Wong *et al *[11,25]. It was expected that knowledge and attitudes would change over time after attending the training program and such a low baseline offered a large room for improvement. The findings largely fulfilled these expectations. For recognition of a mental health problem and reduction of social distance, the effects of this study are promising. By contrast, for the schizophrenia vignette, the level of acceptance of the helpfulness of treatments was found to be lower than for the English- speaking community.

There are some possible reasons for this. First, there is a great deal of personal and social stigma attached to the schizophrenia label in the Chinese community [18]. In Chinese culture, severe mental illness is characterized by two different states: *Dian*, which is a psychotic condition without excitation, and *Kuang*, which is a psychotic condition with excitation [31]. These two psychotic states are both perceived to be signs of madness, with *Kuang *in particular denoting a sense of unpredictability, dangerousness and bizarre and uncontrollable behaviour. Chinese people perceive schizophrenia very negatively and it is not uncommon for Chinese to deny the existence of psychotic illness and to use alternative more socially acceptable labels to describe a condition [30,32]. This denial or avoidance drives the general public away from people with mental illness [33] and limits the participants' understanding of the schizophrenia vignette and the helpfulness of the treatment. Second, the method of teaching may affect the results. MHFA is designed to be a standardized 12 hour training program. However, the instructor for these three courses had to spend a comparatively longer time in teaching and doing role plays for helping people with depression or depression with suicidal thoughts. Hence, the data are more likely to find improvements in depression literacy. Last but not least, programs were conducted in Cantonese and were instantaneously translated into Mandarin. Although both are languages of Chinese, Cantonese is mainly spoken in Guangdong, the southern part of mainland China. People can generally understand Mandarin or Cantonese through writing, but the pronunciation of the two are totally different. This may have affected the understanding of the program by Mandarin speaking participants and hence influenced the results of the study.

These points raise important concerns for the teaching of MHFA and its evaluation in Chinese communities and in other cultural groups. As a training program that originated in English, it is important to play attention to the contextual and cultural differences when adapting the program to another country or culture. Given the negative connotations of traditional Chinese labels for psychotic disorders, particular attention needs to be given to reducing the stigma of these disorders, e.g. by promoting personal contact with a person who has recovered from a psychotic disorder, since contact is known to reduce stigma [34]. In addition, although MHFA is a standardized psychoeducation program, variation in instructor background and teaching style might have an influence on the outcome of the program.

## Limitations

The present study found that MHFA training can improve mental health literacy, which is consistent with the conclusions drawn from a review of evaluation studies on the immediate impact of MHFA training [28,29]. However, longer-term follow-up data are required to find out whether the changes are sustained, and whether the new knowledge and skills will be used appropriately (or at all). Reports from evaluations carried out with English speaking populations show sustained changes in knowledge and attitudes 5-6 months post-training and continued improved confidence in offering assistance. These are questions awaiting further studies in Chinese-speaking immigrant communities.

To allow comparison across studies, it is important to use the same evaluation questionnaires as used in the earlier studies with English-speaking Australians. However, there are potential limitations to the cultural and language transportability of questionnaires. Subtleties of meaning and cultural factors may have influenced the results of the present study, and thus their interpretation when filling a translated questionnaire.

Another limitation is the sample selection, which was chosen for convenience and may not be representative of the broader Australian Chinese community.

Finally, the present study was uncontrolled, so we do not know to what extent any changes are due to the passage of time or re-testing with the questionnaires. A randomized controlled study would be the next step, as has been done in the mainstream Australian community.

## Conclusion

For the Chinese community in Melbourne, MHFA training has been shown to be effective in improving recognition of mental disorders, in reducing negative stigmatizing attitudes and in changing beliefs about the helpfulness of treatment. The evidence from this study, together with the accumulated evidences of the benefits of MHFA training in the general Australian community, suggest that this approach should be scaled up to a level where it can have an impact on the whole of the Chinese community in Australia. Such a scaled up population mental health program should be evaluated over time.

## Competing interests

The second author was the developer of the Mental Health First Aid course.

## Authors' contributions

AYKL co-designed the study and the translation of the evaluation questionnaire, analyzed the data, taught the Mental Health First Aid courses, and wrote the final version of the paper. AFJ designed the English evaluation questionnaire, and co-wrote the manuscript and contributed to the data analysis. DFKW co-designed the study and the translation of the evaluation questionnaire, and contributed to the data analysis. All authors read and approved the final manuscript.
